# Computational Analysis Predicts Hundreds of Coding lncRNAs in Zebrafish

**DOI:** 10.3390/biology10050371

**Published:** 2021-04-26

**Authors:** Shital Kumar Mishra, Han Wang

**Affiliations:** 1 Center for Circadian Clocks, Soochow University, Suzhou 215123, China; mishrasz@suda.edu.cn; 2 School of Biology & Basic Medical Sciences, Medical College, Soochow University, Suzhou 215123, China

**Keywords:** lncRNAs, coding probabilities, bioinformatics, zebrafish

## Abstract

**Simple Summary:**

Noncoding RNAs (ncRNAs) regulate a variety of fundamental life processes such as development, physiology, metabolism and circadian rhythmicity. RNA-sequencing (RNA-seq) technology has facilitated the sequencing of the whole transcriptome, thereby capturing and quantifying the dynamism of transcriptome-wide RNA expression profiles. However, much remains unrevealed in the huge noncoding RNA datasets that require further bioinformatic analysis. In this study, we applied six bioinformatic tools to investigate coding potentials of approximately 21,000 lncRNAs. A total of 313 lncRNAs are predicted to be coded by all the six tools. Our findings provide insights into the regulatory roles of lncRNAs and set the stage for the functional investigation of these lncRNAs and their encoded micropeptides.

**Abstract:**

Recent studies have demonstrated that numerous long noncoding RNAs (ncRNAs having more than 200 nucleotide base pairs (lncRNAs)) actually encode functional micropeptides, which likely represents the next regulatory biology frontier. Thus, identification of coding lncRNAs from ever-increasing lncRNA databases would be a bioinformatic challenge. Here we employed the Coding Potential Alignment Tool (CPAT), Coding Potential Calculator 2 (CPC2), LGC web server, Coding-Non-Coding Identifying Tool (CNIT), RNAsamba, and MicroPeptide identification tool (MiPepid) to analyze approximately 21,000 zebrafish lncRNAs and computationally to identify 2730–6676 zebrafish lncRNAs with high coding potentials, including 313 coding lncRNAs predicted by all the six bioinformatic tools. We also compared the sensitivity and specificity of these six bioinformatic tools for identifying lncRNAs with coding potentials and summarized their strengths and weaknesses. These predicted zebrafish coding lncRNAs set the stage for further experimental studies.

## 1. Introduction

The classical view of the central dogma of molecular biology suggests that DNA makes RNA, and RNA makes proteins. The messenger RNAs (mRNAs) code for proteins by conveying genetic information from the DNA. Subsequently, mRNAs are translated into a polymer of amino acids, the building blocks of a protein. However, many exceptions to this central dogma have been revealed in recent years [1,2]. Only approximately 1% of the whole length of a transcriptome encodes for proteins, and much of its nonprotein-coding region encodes various types of functional RNAs [3]. Among them, the noncoding RNAs longer than 200 nucleotide base pairs are termed long-noncoding RNAs (lncRNAs) [4]. This somewhat arbitrary limit distinguishes lncRNAs from the small noncoding RNAs (sRNAs) [5]. Although lncRNAs do not code for proteins, they play regulatory roles in important biological processes, such as immune response [6,7], cellular growth and development [8].

Intriguingly, technological advancements such as mass spectrometry and ribosome profiling (Ribo-seq) have confirmed that an increasing number of noncoding RNAs (ncRNAs) actually encode functional micropeptides [9,10,11,12]. For example, a recent study [13] used ribosome profiling to identify several micropeptides outside of canonical coding sequences (CDS). This study found hundreds of non-canonical CDSs involved in phenotypic responses and cellular growth. Specifically, many ncRNAs act like proteins to regulate biochemical reactions, and some are suitable to act as scaffolds for molecular interactions [14]. Numerous lncRNAs contain short open-reading frames (sORFs) [15], which can encode micropeptides involved in biologically significant processes such as cellular division and cell signaling [16]. Recent studies found a 90-residue polypeptide encoded by a sORF containing lncRNA LINC00961 [17]. The lncRNA LINC00961-encoded SPAR (Small regulatory polypeptide of amino acid response) is involved in regulating mammalian target rapamycin complex 1 (mTORC1) and muscle regeneration [18], and in regulating endothelial cell function [15]. In the latter case, LINC00961 and its encoded SPAR, two molecules resulting from the same gene locus, appear to play opposing roles in angiogenesis [15]. Another lncRNA, LINC00908, was confirmed to encode ASRPS (a small regulatory peptide of STAT3), a 60–amino acid-long peptide, which inhibits angiogenesis in triple-negative breast cancer (TNBC) cells [19,20]. Zebrafish Toddler (also known as Apela/Elabela/Ende), a lncRNA-encoded micropeptide, promotes cell movement during gastrulation by activating APJ/Apelin receptor signaling [21]. Thus, identification and characterization of lncRNA-encoded micropeptides represents the next regulatory biology frontier [14,20,22,23].

Further, genomic studies have indicated that lncRNAs are involved in regulating genomic transcription [22], human diseases [23], epigenetic and gene regulatory functions [24]. These observations have inspired the genomic researchers to explore the regulatory functions of noncoding genes at the systems level. Rapid development of high-throughput sequencing technology has led to the identification of a huge number of lncRNAs [25], which has tremendously transformed our knowledge of genomic interactions involving ncRNAs. While the wet-lab experiments consume a significant amount of time to obtain solid research results, analyzing the huge amount of sequencing data also requires vast computational resources. With the rapid growth of computing power, it is desirable to integrate computational techniques with experimental observations to enhance the quality of research outcomes.

Despite recent progress in predicting the functions of non-coding RNA genes, our understanding of the computational identification of coding lncRNAs is rather far from complete. In particular, distinguishing coding and noncoding RNAs remains a challenge due to the lack of information for conserved regions, whole-genome sequences and underlying computational difficulties. Bioinformatic analysis driven by high computing power provides a viable approach to classify noncoding RNA genes and expedite the functional genomic research. For example, a recent study [26] reviewed several bioinformatics tools (such as CPC2, PORTRAIT, CNCI, CPAT) and highlighted their strengths and weaknesses. However, to date, none of the tools had been applied to predict the micropeptide encoding probability of a large dataset of zebrafish lncRNAs. Here we employed six bioinformatic tools––Coding Potential Alignment Tool (CPAT), Coding Potential Calculator 2 (CPC2), LGC web server, Coding-Non-Coding Identifying Tool (CNIT), RNAsamba, and MicroPeptide identification tool (MiPepid––to analyze approximately 21,000 zebrafish lncRNAs and computationally identify 2730–6676 with high encoding potential. Of these, 313 coding lncRNAs were predicted by all six bioinformatic tools.

## 2. Materials and Methods

In this study, we employed an integrative approach that combined both computational and data-driven modelling approaches, which was a novel framework for investigating noncoding genes. In particular, we applied six state-of-the-art bioinformatic suites: CPAT [27], CPC2 [28,29], LGC web server [30], CNIT, RNAsamba [31], and MiPepid [32] (Table 1) to classify more than 21,000 lncRNAs collected from the ZFLNC [33], Ensembl [34], NONCODE [35], and zflncRNApedia [36] databases.

### 2.1. Rationale for Selection of the Six Bioinformatic Tools

We selected these six tools for the following reasons. CPAT is an alignment-free, robust, logistic-regression model that can integrate prior knowledge of DNA sequences. The tool is applicable to four species: humans, mice, zebrafish and fruit flies. Furthermore, it predicts various useful genomic measures, such as RNA size, ORF size, and coding probability, Fickett score and Hexamer score. CPC2 is a support vector machine (SVM)-based species-neutral fast RNA classifying tool that provides genomic annotation information, such as ORF length, ORF position, peptide length, Fickett score, and coding probability. The LGC web server classifies lncRNAs by establishing feature relationships between guanine–cytosine (GC) content and ORF length, so the server can classify genomic sequences in a cross-species manner without depending on any prior knowledge. The prediction output of the web server includes the GC content of the longest ORF, coding potential score, coding label, probability of ORF in a coding sequence, probability of ORF in a non-coding sequence, stop-codon probability for a coding sequence, and stop-codon probability in a coding sequence. CNIT predicts coding probabilities by analyzing the composition of adjoining nucleotide triplets (ANTs) in the sequences. The tool is effective for predicting the coding labels of incomplete transcripts and sense-antisense transcript pairs. Further, CNIT can assess the coding potential of lncRNAs from 37 species: 11 animal and 26 plant. The output of CNIT includes both the coding label and coding score. RNAsamba analyzes novel genomic sequences using a neural network-based model framework to predict coding potential. The tool efficiently predicts small ORFs, which is otherwise often identified with time-consuming biological experiments such as ribosome profiling. Moreover, RNAsamba can predict the coding potentials of incomplete transcripts, such as partial-length ORFs and UTR sequences. MiPepid is an alignment-free, machine-learning tool specifically developed to identify micropeptides. The tool predicts coding potential using a logistic regression with 4-mer features. For each lncRNA sequence, MiPepid can identify all the sORFs 3–303 nucleotides long, and predict the coding potential for each identified sORF. Further, except for MiPepid, the other five tools can be accessed on the web. Overall, we believe that these six tools enable a very comprehensive analysis of lncRNAs.

### 2.2. Robustness of the Bioinformatic Tools

In order to statistically measure the validity of the predictions made by bioinformatic tools to identify coding lncRNAs, we assessed the gold-standard sensitivity and specificity measures [38] for the tools. The sensitivity measure assessed the ability of a tool to predict lncRNA coding potentials correctly, whereas specificity measured the ability of a tool to identify lncRNAs without a coding potential. Since the previous study [27], had already assessed to CPAT have a sensitivity and specificity of 0.96, and 0.97, respectively (with very limited scope for further improvement) we used it as a benchmark tool and assessed the sensitivity and specificity of the rest of the five tools in this study. The statistical measures were calculated as mentioned below:

Sensitivity = TP/(TP + FN)

Specificity = TN/(TN + FP)

where TP is the number of true positives, TN is the number of true negatives, FN is the number of false negative, and FP is the number of false positives. Specifically, for each of the 21,128 lncRNAs, TP represented the coding potentials when both the CPAT and the tool being compared predicted a lncRNA as coding; FN when the CPAT predicted a lncRNA as coding and the tool being compared predicted a lncRNA as noncoding; TN represented the coding potentials when both the CPAT and the tool being compared predicted a lncRNA as noncoding; and FP when the CPAT predicted a lncRNA as noncoding and the tool being compared predicted a lncRNA as coding.

## 3. Results

Identification of coding lncRNAs from a 21,000 zebrafish lncRNA database with 6 bioinformatic tools.

### 3.1. Coding Potential Alignment Tool (CPAT)

Predicting the coding probability of a genomic sequence is a binary decision-making problem. An interesting tool to predict the coding probability is the alignment-free Coding Potential Alignment Tool (CPAT) [27]. CPAT is suitable for assessing the coding probabilities of unknown lncRNA sequences, the functions of which are still being investigated. As long as the sequencing information of an lncRNA is available, the CPAT can be used to examine its coding probability. The tool accepts standard FASTA-format input sequences, analyzes them based on trained models, and outputs the coding probabilities. The software also provides other statistical measures for sequences, such as RNA size, ORF size, Fickett Score core and Hexamer Score. The combined feature set has a sensitivity and specificity of 0.96 and 0.97, respectively. Such a high degree of prediction accuracy for the CPAT in discriminating a coding from noncoding sequence was achieved using a logistic regression model. The CPAT not only outperformed the CPC [28] (assessed in the following subsection), but it was four times faster. Moreover, due to very high sensitivity and specificity, there was only a little room for further improvement. A recent study [39] found that the CPAT distinguished between coding and noncoding mammalian transcripts in the most accurate manner. We applied the CPAT to predict the coding probabilities for the zebrafish lncRNAs from the ZFLNC lncRNAs database [33]. Out of 21,128 lncRNAs, 4386 sequences were predicted to have a very high coding potential (Supplementary Tables S1 and S7). However, the tool only accepted input files smaller than 10 MB. Hence, we divided the 21,128 lncRNA input sequences in multiple files. Overall, the CPAT took less than 3 min to estimate the coding probabilities for all 21,128 lncRNAs. Supplementary Table S1 shows a sample output of the CPAT for the ZFLNC input sequences. Taken together, the CPAT is a robust, fast, and convenient tool for investigating the novel transcript sequences.

### 3.2. Coding Potential Calculator 2 (CPC2)

The Coding Potential Calculator 1 (CPC1) [28] tool was one of the earliest software suites to distinguish between coding and noncoding RNAs. Essentially, the tool predicted the protein-coding potential of a given cDNA/RNA transcript. It classified the FASTA RNA sequences using the support-vector machine and six biological features (such as the Open Reading Frame) of a given input transcript. Based on a tenfold cross-validation, CPC1 was assessed to classify the RNAs with a very high level of accuracy and speed. Its sensitivity and specificity were 0.99 and 0.74, respectively. However, classification of the RNA sequences based on the ORF often met with several limitations. For example, the ORF length requires assembly of the full-length transcript. Moreover, since a novel-assembled RNA transcript may be still be incomplete, it could be challenging to use CPC1 for RNA genes that have incomplete transcripts. Subsequently, CPC1 was thoroughly revised and updated to become Coding Potential Calculator 2 (CPC2) [29]. Compared to CPC1, it can distinguish between coding and noncoding sequences with an improved accuracy at ∼1000 × faster speed and can accept both individual and batch input sequences. Moreover, the underlying model of CPC2 is species-neutral. Hence, it is suitable for assessing the coding potential of the rapidly emerging demands of non-model organism sequences. We used CPC2 to distinguish the coding probabilities of 21,128 zebrafish lncRNAs. Out of the 21,128 sequences, 2370 sequences were predicted to be “coding” sequences (Supplementary Tables S2 and S8). It was also able to classify the 21,128 lncRNAs with a sensitivity of 0.5709 and a specificity of 0.9865. Further, CPC2 predicted other genomic measures such as the putative peptide length, isoelectric point (pI), and Fickett test code score. For example, CPC2 gave sequence ZFLNCT00001 a Fickett score of 0.44008 with a complete putative ORF 101 AA, and a pI of 10.9258422852, which classified it as a coding sequence with coding probability 0.5. However, for sequence ZFLNCT00002, CPC2 predicted a Fickett score of 0.32995 with a complete putative ORF 54 AA, and a pI of 4.20184326172, which classified it as a noncoding sequence with a coding probability of 0.0513828. Although CPC2 also required input files constrained to a maximum of 10,000 sequences and a file size smaller than 50 Mb, all input files containing 21,128 lncRNAs were processed in less than 2 min. Supplementary Table S2 shows a sample output of CPC2 for ZFLNC sequences. Overall, the species-neutral model of CPC2 makes it useful for investigating the poorly annotated novel sequences from a variety of organisms. The CPC2 web server provides online visualization of the results and features of the sequences, such as Fickett score and ORF length. Further, the improved accuracy and mobile-friendly version of CPC2 makes it suitable for investigating ever-growing sequences from the non-model organisms. However, despite CPC2’s higher specificity (0.9865) for novel zebrafish lncRNAs, the lower sensitivity (0.5709) of the tool suggests that it may require further calibration to improve prediction sensitivity.

### 3.3. LGC Web Server

The LGC web server [30] was developed to distinguish the lncRNAs and protein coding genes in a cross-species manner covering various species from plants to mammals without any prior knowledge. The tool assesses the relationship between open reading frame length and guanine–cytosine (GC) content to predict the coding potentials of the given genomic sequences. The server accepts FASTA, BED and GTF format input sequences and predicts multiple genomic features:

1. ORF Length (length of the longest ORF),
2. GC Content (GC content of the longest ORF),
3. Coding Potential Score for the transcript (protein-coding RNA if greater than 0 or ncRNA if smaller than 0),
4. Coding Label (Coding and Non-coding),
5. The probability of ORF for coding sequence (p_c_),
6. The probability of ORF for non-coding sequence (p_nc_),
7. Stop-codon probability for coding sequence (f_c_), and
8. Stop-codon probability for non-coding sequence (f_nc_).

The proposed LGS algorithm outperformed existing methods for accuracy, sensitivity, and specificity. Moreover, this is the first tool to accurately differentiate noncoding and coding sequences based on a feature relationship between ORF length and GC content. The main advantage of the LGC web server lies in its ability to process and classify sequences from a diverse set of species without the need for any prior knowledge or training data.

When we applied the LGC web server to ZFLNC sequences, 3156 lncRNA sequences were predicted to have very high coding abilities (Supplementary Tables S3 and S9). The server was able to classify the 21,128 lncRNAs with a sensitivity of 0.4591 and a specificity of 0.9317. Further, the LGC web server was able to process the whole file, a feature better than other tools that imposed file size restrictions. However, the server took nearly 10 min to process the whole set of lncRNA sequences. Supplementary Table S3 shows the LGC web server’s sample output of coding potential score calculation.

Despite the tool’s higher specificity in classifying lncRNAs without coding potential, there remains a higher scope for improving its sensitivity for the novel zebrafish lncRNAs. Compared to the CPAT sequence analysis (Supplementary Table S1), the LGC also accurately predicted the ORF lengths for the zebrafish noncoding RNAs. However, there are significant differences regarding coding potentials and coding labels. The main reason behind these differences is that CPAT can specifically process zebrafish sequences. In fact, LGC accepts species-specific sequence inputs. Moreover, despite achieving higher accuracy (>90%), it is not yet established if the LGC web server could be applied to investigate the small noncoding RNAs.

### 3.4. Coding–Non-Coding Identifying Tool (CNIT)

Coding–Non-Coding Index (CNCI) software [40] was developed to classify incomplete transcripts and sense-antisense pairs, obtained from whole-genome sequencing data, into coding and noncoding transcripts. The inputs to CNCI are the transcripts derived from the whole-transcriptome sequencing. Subsequently, CNCI analyzes adjoining nucleotide triplets using sequence-specific information to make the sequence classification without prior knowledge of a transcript annotation. Since the CNCI tool uses only sequence-specific features, it can be used to classify novel genomic sequences from a variety of species without the availability of whole-genome sequences. Although CNCI is good for classifying incomplete transcripts (even in a cross-species manner) and performed well with vertebrates, its performance with plants and invertebrates was relatively poor. Moreover, CNCI only analyzes lncRNAs longer than 200 nt, so it is not yet applicable to miRNAs.

To improve computational performance and include more species, CNCI was upgraded with the sequence-intrinsic, features-based Coding–Non-Coding Identifying Tool (CNIT) [37]. Essentially, the CNIT profiles adjoining nucleotide triplets (ANTs) to differentiate between coding and noncoding sequences. The upgraded software is able to functionally classify novel lncRNA sequences with improved accuracy. Moreover, the CNIT runs approximately 200 times faster than the CNCI software and is applicable to sequences from a higher number of species, including 27 plants. In particular, the mobile-friendly web-sever version of the software makes it suitable for predicting the coding potential of a small number of sequences from a variety of species that lack whole-genome sequences.

We applied the CNIT to classify the ZFLNC sequences. It predicted a total of 5651 sequences to be “coding” (Supplementary Tables S4 and S10), and classified the 21,128 lncRNAs with a relatively higher sensitivity of 0.7218 and an acceptable specificity of 0.8515. Further, the CNIT could only process FASTA files smaller than 400 KB. This required the division of the 21,128 lncRNAs into over 100 files, and the CNIT took over two hours to complete the coding ability prediction for all of them. Apart from the slow processing speed, the major issue with the CNIT is the overall lower sensitivity of the classification of the zebrafish lncRNAs.

### 3.5. RNAsamba

RNAsamba [31] is a novel neural network-based framework for predicting the coding potential of genomic sequences by assessing the ORF and other sequencing information. The performance of RNAsamba was evaluated from the on-transcripts from a diverse set of five organisms, including human, *M. musculus*, *D. rerio*, *D. melanogaster* and *Saccharomyces cerevisiae*. RNAsamba outperformed all the tools examined in the study, including the CPAT and CPC2. Moreover, RNAsamba was able to predict coding potentials of incomplete transcript sequences, such as partial-length ORFs and UTR sequences. In fact, RNAsamba could computationally predict open reading frames (sORF) without depending on time-consuming ribosome profiling experiments. Overall, RNAsamba is suitable for classifying novel genomic sequences with enhanced accuracy while improving prediction time. When we applied RNAsamba to the 21,128 lncRNAs, it took just about 2 min to assign a coding score and label to all sequences. A total of 6676 sequences were classified to coding lncRNAs (Supplementary Tables S5 and S11). RNAsamba outperformed the other five state-of-the-art bioinformatic tools by classifying the 21,128 lncRNAs with an acceptable sensitivity of 0.8780 and an acceptable specificity of 0.8321. However, despite fast calculation and improved prediction efficiency, the tool only supports FASTA, FA and FNA file extensions. Furthermore, the input files are restricted to 50 MB and can only contain up to 50,000 sequences.

Since RNAsamba had the best sensitivity and specificity for classifying the zebrafish lncRNAs, we applied it to an investigation of the zebrafish Toddler (NCBI RefSeq transcript identifier NM_001297547.1). Toddler contains nucleotide sequence ORF that encodes a 58 amino acid-long conserved micropeptide (Supplementary Table S12) with a confirmed predicted signal sequence [21]. We wanted to validate if RNAsamba could accurately classify the Toddler ORF. Interestingly, RNAsamba classified the ORF as “coding” with a coding probability of 0.65645. This analysis further supported our assessment that RNAsamba can reliably classify zebrafish lncRNAs.

### 3.6. MicroPeptide Identification Tool (MiPepid)

Micropeptides are the short open reading frames (sORF) that encode small proteins containing approximately 100 amino acids. Due to technical challenges [41], sORF-encoded micropeptides have been traditionally excluded from genomic studies [42]. Recent studies have reported an increasing number of micropeptides involved in a diverse set of biological functions. However, we still lack sophisticated tools to find micropeptide-encoding potentials of the novel sequences. The MiPepid tool [32] was designed to assess micropeptides encoding potentials of DNA sequences by investigating the presence of short open reading frames. The tool analyses DNA sequences, finds all the sORF, and predicts micropeptide-encoding probability for each sORF. When we applied MiPepid to investigate sORF-encoded micropeptides in ZFLNC sequences, the tool identified 186,504 micropeptides in 4676 lncRNAs (Supplementary Table S13). As many as 126,175 micropeptides were reported to have coding potential. Some of these micropeptides were encoded by only 6 nucleotides, whereas others were encoded by as many as 303. We classified a particular lncRNAs sequence as coding if it contained at least one 100-nucleotide-long sORF with a “coding” label. Overall, out of 4676 lncRNAs, 3786 were classified as coding lncRNAs (Supplementary Tables S6 and S14). MiPepid was able to classify the 21,128 lncRNAs with the lowest sensitivity (0.1931) and an acceptable specificity (0.8244). Although it is suitable for predicting sORF-encoded micropeptides, MiPepid can only predict coding potential for sequences having a nucleotide length between 6 and 303. In fact, MiPepid cannot process RNA sequences. Furthermore, letters such as N, R, Y, are not supported in the current version of MiPepid, and as such it needs to be upgraded to investigate ORF sequences longer than 303.

### 3.7. Robustness of the Prediction of Bioinformatic Tools

Table 2 shows the sensitivity and specificity for the 5 bioinformatic tools, in comparison with the CPAT. The study revealed that all 5 demonstrated acceptable specificity; however, only RNAsamba had very high sensitivity (0.8780) and specificity (0.8312). Hence, in addition to the CPAT, RNAsamba is a better tool for identifying zebrafish coding lncRNAs than other four.

## 4. Discussion

**Figure 1:**
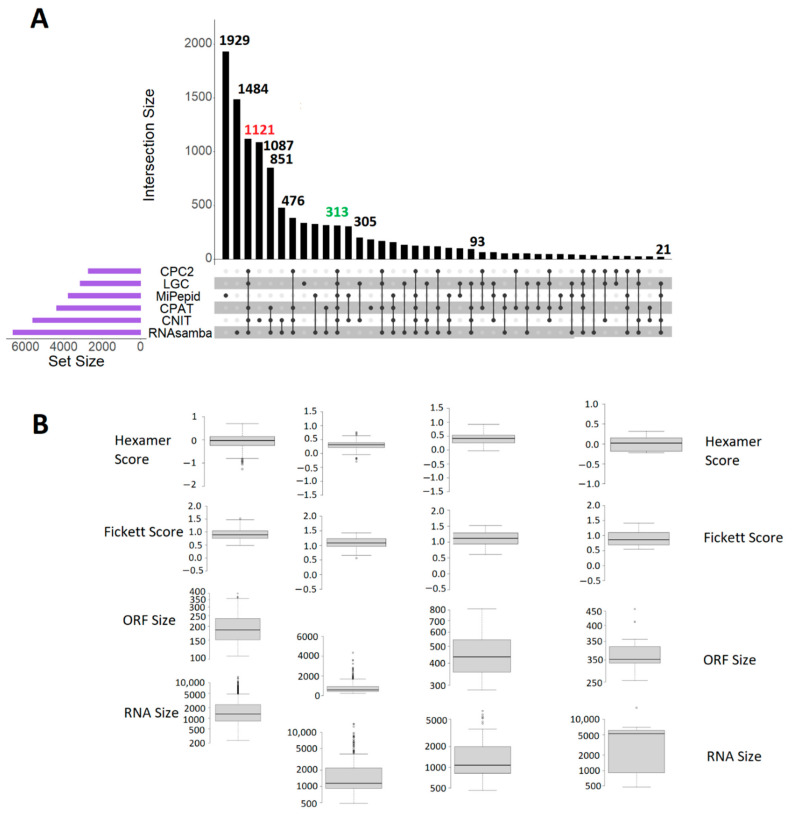
Visualization of the number of coding lncRNAs, and box-plot statistics (Supplementary Table S17) of RNA size, ORF size Fickett Score, and Hexamer score for 1, 929, 313, 93, and 21 sequences predicted to be coding by the corresponding bioinformatic tools. Out of the 21,128 lncRNAs examined in this study, 313 (green) were predicted (Supplementary Table S16) to be coding by all 6 tools (**A**). The box-plots depict the upper whisker (represented by the bubbles on the top), 3rd quartile, median, 1st quartile, and lower whisker (represented by the bubbles at the bottom) for the given number of data points (**B**).

Over the past few decades, the investigation and understanding of noncoding RNAs have drawn unprecedented attention from the scientific research community for their roles in gene regulation [8] and in a variety of human diseases such as cancers [19,20]. Differentiating coding and noncoding RNA transcripts is often constrained by time-consuming biological research and computational limitations. However, the emergence of high-throughput sequencing has facilitated a deeper investigation into noncoding RNAs. Here, we compared several recent software suites to analyze, classify, and annotate the noncoding RNAs by combining sequencing data and computational mechanisms. We calculated the coding potentials of over 21,000 zebrafish lncRNAs using the CPAT, CPC2, LGC web server, the CNIT, RNAsamba, and MiPepid and revealed 4386, 2730, 3156, 5651, 6676 and 3786 lncRNAs with very high coding potentials, respectively. In particular, we found 313 lncRNAs predicted to be “coding” according to all six bioinformatic tools (Figure 1, and Supplementary Table S15).

In fact, as many as 1121 lncRNAs were predicted to be coding by five tools (excluding MiPepid) (Figure 1). The main reason behind the poor overlap with other five is MiPepid’s restriction of ORF size between 6 and 303 nucleotides. However, the rest of the five tools exploited the sequence-intrinsic features to provide a relatively higher number of overlapping predictions. Of the six tools, the CPC2 was the fastest for predicting the coding abilities of the novel lncRNAs. The CNIT was the slowest for classifying the RNA sequence, taking more than two hours to complete the computation. Although all tools accepted a batch job submission, only the LGC web server was able to process the 21,128 lncRNAs in a single job. The remaining five tools required input sequences split over multiple files.

Although the six tools were suitable for analyzing genomic sequences, they were far from perfect. For example, apart from the LCG web server, all the others restricted the size of input files, typically from 10 to 50 MB. Such limitations will impose challenges to process sequencing files of larger size. The tools need to be upgraded to consider species-specific information. Further, the LCG web server assesses coding potentials without considering species-specific information. The CPAT supports only a limited number of species, namely Human (hg19, GRCh37), Mouse (NCBI Build 37/mm9), Mouse (GRCm38/mm10), Fly (dm3, BDGP Release 5), and Zebrafish (Zv9/danRer7). The CNIT is able to support the maximum number of 37 species. In fact, only RNAsamba was able to correctly classify the Toddler ORF as “coding”. As such, all these bioinformatics tools will need to be upgraded as sequencing information for the new species become available.

Such functional classification can help annotate lncRNAs to reveal their unknown functions, which should show how RNAs interact with other coding RNAs. Integrative research approaches, which combines both computational and data-driven modelling, represent a novel paradigm for investigating noncoding RNAs at the system level. These computational analyses can help uncover novel coding RNAs that may play roles in gene regulation or the pathogenesis of various human diseases. We believe, as more data and computational tools become available, the integrative framework will be suitable to further elucidate crucial lncRNA functions.

## 5. Conclusions

The regulatory roles of lncRNAs and particularly lncRNA-encoded micropeptides have not yet fully explored. It is critically important to develop and implement bioinformatic tools for identifying coding lncRNAs. Here, we evaluated six bioinformatic programs for computing coding probabilities of noncoding RNAs, and discussed their strengths and shortcomings as well as their possible improvements. We also employed these six bioinformatic tools to identify several thousands of zebrafish coding lncRNAs, which should set the stage for their functional characterization of them.

## Figures and Tables

**Table 1:** List of computational tools for noncoding RNA analysis (accessed on 4 February 2021).

Software Suite	Journals Publishing the Software	Year	Applied Species	Input	Output	URLs
Coding Potential Alignment Tool (CPAT) [27]	Nucleic Acids Research	2013	*Homo sapiens*, *Mus musculus*, *Danio rerio, Drosophila melanogaster*	BED, FASTA	Coding Probability, ORF size, Fickett Score, Hexamer Score, Coding Label	http://lilab.research.bcm.edu/
Coding Potential Calculator 2 (CPC2) [28,29]	Nucleic Acids Research	2017	*Homo sapiens*, *Mus musculus*, *Xenopus laevis*, *Danio rerio* and *Drosophila melanogaster*	FASTA, BED or GTF	Peptide length, Fickett score, Pi, ORF integrity, coding probability, coding label	http://cpc2.gao-lab.org/ http://cpc2.gao-lab.org/batch.php
LGC web server [30]	Bioinformatics	2019	Cross-species manner from plants to mammals	FASTA, BED, GTF,	ORF Length, Coding Potential Score for the transcript, Coding/Non-coding Label, Probability of ORF for coding sequence (pc),	https://bigd.big.ac.cn/lgc/calculator
Coding-Non-Coding Identifying Tool (CNIT) [37]	Nucleic Acids Research	2019	37 species (11 animal species, 26 plant species)	FASTA, GTF	Gene classification (coding or noncoding)	http://cnit.noncode.org/CNIT/ http://cnit.noncode.org/CNIT/batch
RNAsamba [31]	NAR Genomics and Bioinformatics	2019	*Mus musculus*, *Danio rerio*, *Drosophila melanogaster*, *Caenorhabditis elegans* and *Arabidopsis thaliana*	FASTA, FA and FNA	Coding score and classification	https://rnasamba.lge.ibi.unicamp.br/
MicroPeptide identification tool (MiPepid) [32]	BMC Bioinformatics volume	2019	*Mus musculus*, *Danio rerio, Saccharomyces cerevisiae*, *E. coli* and *Arabidopsis thaliana*	FASTA		https://github.com/MindAI/MiPepid

**Table 2:** Sensitivity and specificity of the computational tools *.

Robustness	CPC2	LGC	CNIT	RNAsamba	MiPepid
Sensitivity	0.5709	0.4591	0.7218	0.8780	0.1931
Specificity	0.9865	0.9317	0.8515	0.8312	0.8244

* In the Table, CPC2, Coding Potential Calculator 2; LGC, LGC web server; CNIT, Coding-Non-Coding Identifying Tool; RNAsamba; and MiPepid, MicroPeptide identification tool.

## Data Availability

The zebrafish lncRNA sequences can be found at http://www.zflnc.org/download (accessed on 4 February 2021). All the coding potential data generated in this study can be found in the supplementary materials.

## Supplementary Materials

The following are available online at https://www.mdpi.com/article/10.3390/biology10050371/s1, Table S1. Sample output from the analysis of zebrafish lncRNAs with CPAT; Table S2. Sample output from the analysis of zebrafish lncRNAs with CPC2; Table S3. Sample output from the analysis of novel ZFLNC lncRNAs with LGC web server; Table S4. Sample output from the analysis of zebrafish lncRNAs with CNIT; Table S5. Sample output from the analysis of zebrafish lncRNAs with RNAsamba; Table S6. Sample output from the analysis of zebrafish lncRNAs with MiPepid; Table S7. Coding probabilities of 21, 218 lncRNAs predicted by CPAT; Table S8. Coding probabilities of 21, 218 lncRNAs predicted by CPC2; Table S9. Coding probabilities of 21, 218 lncRNAs predicted by LGC Web Server.; Table S10. Coding probabilities of 21, 218 lncRNAs predicted by CNIT; Table S11. Coding probabilities of 21, 218 lncRNAs predicted by RNAsamba; Table S12. Analysis of zebrafish Toddler sequence; Table S13. Coding probabilities of 21, 218 lncRNAs predicted by MiPepid; Table S14. Coding probabilities of 21, 218 lncRNAs predicted by MiPepid; Table S15. Comparisons of coding potentials of 21, 218 lncRNAs predicted by all the six tools; Table S16. 313 lncRNAs predicted to be coding by all the six tools; Table S17. Box plot statistics of the selected groups of coding lncRNAs predicted by these bioinformatic tools.
