# Supplementary material for: Computational Analysis Predicts Hundreds of Coding lncRNAs in Zebrafish
Source: Biology (Basel). 2021 Apr 26;10(5):371. doi: 10.3390/biology10050371 (PMC8145020; doi:10.3390/biology10050371)
Supplement: Supplementary file 1 [file biology-10-00371-s001.zip › biology-1173552-supplementary/biology-1173552-supplementary for conversion.pdf]

Table S1. Sample output from the analysis of zebrafish lncRNAs with CPAT.

Sequence Name	RNA size	ORF size	Fickett Score	Hexamer Score	Coding probability	Coding Label
ZFLNCT00001	482	303	0.9357	0.122997258	0.484877418	yes
ZFLNCT00002	907	162	1.182	0.538316165	0.13260825	no
ZFLNCT00003	708	162	1.182	0.538316165	0.153116057	no
ZFLNCT00004	632	114	0.8425	−0.071883631	0.000306917	no
ZFLNCT00005	1012	810	1.3009	0.542508917	0.999999997	yes
ZFLNCT00006	847	363	1.2835	0.463472239	0.985139072	yes
ZFLNCT00007	218	60	1.1063	0.062158952	0.000172059	no
ZFLNCT00008	534	354	1.1932	0.824525167	0.999315038	yes
ZFLNCT00009	852	660	1.1299	0.516208644	0.999999606	yes
ZFLNCT00010	2708	414	0.8459	−0.445605543	0.051020498	no

Table S2. Sample output from the analysis of zebrafish lncRNAs with CPC2.

#ID	Peptide Length	Fickett Score	PI	ORF Integrity	Coding Probability	Coding Label
ZFLNCT00001	101	0.440	10.925	1	0.5	coding
ZFLNCT00002	54	0.329	4.201	1	0.0513828	noncoding
ZFLNCT00003	54	0.438	4.201	1	0.132773	noncoding
ZFLNCT00004	38	0.381	10.044	1	0.0192227	noncoding
ZFLNCT00005	270	0.436	8.601	1	0.99993	coding
ZFLNCT00006	121	0.463	8.719	−1	0.11155	noncoding
ZFLNCT00007	20	0.346	5.962	1	0.00366518	noncoding
ZFLNCT00008	118	0.458	6.327	−1	0.0698773	noncoding
ZFLNCT00009	220	0.474	5.089	1	0.999849	coding
ZFLNCT00010	138	0.327	9.877	1	0.360331	noncoding

Table S3. Sample output from the analysis of novel ZFLNC lncRNAs with LGC web server.

# Sequence Name	ORF Length	GC Content	Coding Potential Score	Coding Label	pc	pnc	fc	fnc
ZFLNCT00001	303	0.554	−0.518	Non-coding	1.71E − 05	2.86E − 05	0.006	0.014
ZFLNCT00002	162	0.574	−0.818	Non-coding	2.26E − 05	5.13E − 05	0.006	0.013
ZFLNCT00003	162	0.574	−0.287	Non-coding	3.92E − 05	5.23E − 05	0.006	0.013
ZFLNCT00004	114	0.482	−0.495	Non-coding	5.29E − 03	8.68E − 03	0.007	0.015
ZFLNCT00005	810	0.531	1.157	Coding	4.48E − 06	1.41E − 06	0.006	0.014
ZFLNCT00006	261	0.517	−0.769	Non-coding	7.52E − 08	1.62E − 07	0.007	0.015
ZFLNCT00007	0	0.00E + 00	0.00E + 00	Non-coding	1.00E + 00	1.00E + 00	1	1
ZFLNCT00008	165	0.539	−0.369	Non-coding	4.54E − 03	6.56E − 03	0.006	0.014
ZFLNCT00009	660	0.573	0.357	Coding	8.24E − 06	5.77E − 06	0.006	0.013
ZFLNCT00010	414	0.459	0.335	Coding	4.37E − 08	3.12E − 08	0.007	0.016

Table S4. Sample output from the analysis of zebrafish lncRNAs with CNIT.

Sequence Name	Transcript ID	Index	CNIT Score
ZFLNCT00001	ZFLNCT00001 gene = ZFLNCG00001	noncoding	−0.346130008
ZFLNCT00002	ZFLNCT00002 gene = ZFLNCG00002	coding	0.744159421
ZFLNCT00003	ZFLNCT00003 gene = ZFLNCG00002	coding	0.693137141

ZFLNCT00004	ZFLNCT00004 gene = ZFLNCG00003	noncoding	−0.406157522
ZFLNCT00005	ZFLNCT00005 gene = ZFLNCG00004	coding	0.798780646
ZFLNCT00006	ZFLNCT00006 gene = ZFLNCG00004	coding	0.728479448
ZFLNCT00007	ZFLNCT00007 gene = ZFLNCG00005	noncoding	−0.350328732
ZFLNCT00008	ZFLNCT00008 gene = ZFLNCG00006	coding	0.754718356
ZFLNCT00009	ZFLNCT00009 gene = ZFLNCG00007	coding	0.779479185
ZFLNCT00010	ZFLNCT00010 gene = ZFLNCG00008	noncoding	−0.371695682

Table S5. Sample output from the analysis of zebrafish lncRNAs with RNAsamba.

Sequence name	Coding Score	Classification
ZFLNCT00001 gene = ZFLNCG00001	0.0882	noncoding
ZFLNCT00002 gene = ZFLNCG00002	0.89599	coding
ZFLNCT00003 gene = ZFLNCG00002	0.49532	noncoding
ZFLNCT00004 gene = ZFLNCG00003	0.087	noncoding
ZFLNCT00005 gene = ZFLNCG00004	0.99833	coding
ZFLNCT00006 gene = ZFLNCG00004	0.97536	coding
ZFLNCT00007 gene = ZFLNCG00005	0.00422	noncoding
ZFLNCT00008 gene = ZFLNCG00006	0.99391	coding
ZFLNCT00009 gene = ZFLNCG00007	0.99933	coding

Table S6. Sample output from the analysis of zebrafish lncRNAs with MiPepid.

sORF_ID	sORF_seq	Start at	End at	Classification	Probability	Length
ZFLNCT00001_ORF3	ATGTTTCGCGGGCATTCGGTCATAATCAACATCAGTGATCGTAATGAAGCGTAA	47	100	coding	0.999266	54
ZFLNCT00001_ORF4	ATGAAGCGTAATTAA	90	104	coding	0.754274	15
ZFLNCT00001_ORF6	ATGAAGAAGACACAGGGTCTCTGA	414	437	noncoding	0.448668	24
ZFLNCT00002_ORF2	ATGTGA	475	480	noncoding	0.962715	6
ZFLNCT00002_ORF4	ATGGCGAGAGTGTGTGCGGGGGGGCTCTGCTGGAGGGACCCTGGCTGA	700	747	coding	0.995684	48
ZFLNCT00002_ORF6	ATGCAGCTGTGCGGAGGGATACGCTCTGGCGGATGA	527	562	coding	0.999936	36
ZFLNCT00002_ORF7	ATGGCAGGTGCTGATCGACTATAA	677	700	coding	0.999992	24
ZFLNCT00002_ORF8	ATGAGGCCAGTGCGCTTCTCCAGCGCTTCAGACGCGCAAACTCCGGCTTTCTGGAGGAGATGA	75	137	coding	0.99691	63
ZFLNCT00002_ORF9	ATGAGGAGGCGCGCGAGGTGTTCGAGGACGACGACAGGACGAAACAGTTCTGGTTGA	183	239	coding	0.999865	57
ZFLNCT00003_ORF2	ATGCATGAAGAAAGTGACGTATAA	313	336	coding	0.926431	24
ZFLNCT00003_ORF3	ATGAATATGAAAATGTTCCTATAG	457	480	noncoding	0.9999	24
ZFLNCT00003_ORF4	ATGAAAATGTTCCTATAG	463	480	noncoding	0.993778	18
ZFLNCT00003_ORF5	ATGTTCCTATAG	469	480	coding	0.729289	12
ZFLNCT00003_ORF6	ATGAGGCCAGTGCGCTTCTCCAGCGCTTCAGACGCGCAAACTCCGGCTTTCTGGAGGAGATGA	41	103	coding	0.99691	63

ZFLNCT00003_ORF7	ATGAGGAGGCGCGCGAGGTGTTCGAGGACGACGACAGGACGAAACAGTTCTGGTTGA	149	205	coding	0.999865	57
ZFLNCT00003_ORF10	ATGGCAACCGTAGTTTCCTACATAAATCTGAGGGAGATTAAAACACTACAGGTCTAA	249	305	coding	0.60653	57
ZFLNCT00003_ORF11	ATGTGTGTAAATCTATATTTATTCAGTTTTTAA	402	434	noncoding	0.737656	33
ZFLNCT00004_ORF1	ATGACTTTCGTCATTGCGTTATTGATCCACATGATGTATTTGGAGACCTGTGTGTAG	556	612	coding	0.991437	57
ZFLNCT00004_ORF2	ATGATGTATTTGGAGACCTGTGTGTAG	586	612	coding	0.970752	27

ZFLNCT00001_ORF1	ATGACGACGGACTTTCTGAAACCAGTTAACCTTTCATCAGGATACCCGATTAAAACACCATTCCTGCGCTTCTGCTCCTGCCATCGCTGGTCGGGGACGGTGAAGATCTCTCTGGCCTCCTCATATGAGCAGATCTCCTCCAGACACTCGCGCTCCAGATCTCCCAGCTTCAGCTCCTCCAGGAAAGAGTTCGCCCTGCGCACACGCGGATGCAGCGGCCGGTTCGCCTCTGCACGACTCAAGAACACTGCAGATCATGGACACACACACACACACACACACACACACACACACACCGGATGA	ZFLNCT00001	115	417	coding	0.997758	303
ZFLNCT00001_ORF2	ATGAAGTGGGAGGAGTCAATTAGCCCGCGCTCAGTTGTGGCGCGCATGTTTCGCGGGCATTCGGTCATAATCAACATCAGTGATCGTAATGAAGCGTAA	ZFLNCT00001	2	100	coding	0.990576	99
ZFLNCT00001_ORF3	ATGTTTCGCGGGCATTCGGTCATAATCAACATCAGTGATCGTAATGAAGCGTAA	ZFLNCT00001	47	100	coding	0.999266	54
ZFLNCT00001_ORF4	ATGAAGCGTAATTAA	ZFLNCT00001	90	104	coding	0.754274	15
ZFLNCT00001_ORF5	ATGCAGCGGCCGGTTCGCCTCTGCACGACTCAAGAACACTGCAGATCATGGACACACACACACACACACACACACACACACACACACACCGGATGAAGAAGACACAGGGTCTCTGA	ZFLNCT00001	324	437	coding	0.999595	114
ZFLNCT00001_ORF6	ATGAAGAAGACACAGGGTCTCTGA	ZFLNCT00001	414	437	noncoding	0.448668	24
ZFLNCT00002_ORF1	ATGGCAACCGTAGTTTCCTACATAAATCTGAGGGAGATTAAAACACTACAGACAAGGAGCCCTGCTTGA	ZFLNCT00002	283	351	coding	0.759022	69
ZFLNCT00002_ORF2	ATGTGA	ZFLNCT00002	475	480	noncoding	0.962715	6
ZFLNCT00002_ORF3	ATGACGGGACGTCCTGCGTCTCTCAGGTTGATTATCCGTGTGGAAAGATTCCTGTGCAGAAAAACACTTCTCAGAATCAGTTCCTGGGTGGAATTCACTGTCCCAGAGGTCACTGTCCATGGCAGGTGCTGA	ZFLNCT00002	559	690	coding	0.989761	132

Tables S7–S17. are provided separately, attached as an Excel File.
